# Augmented reality with algorithm animation and their effect on students’ emotions

**DOI:** 10.1007/s11042-022-13679-1

**Published:** 2022-09-07

**Authors:** Maximiliano Paredes-Velasco, J. Ángel Velázquez-Iturbide, Mónica Gómez-Ríos

**Affiliations:** 1 Department of Computing and Statistics, Rey Juan Carlos University, C/ Tulipán, s/n, 28933 Móstoles, Madrid, Spain; 2 Department of System Engineering, Salesian Polytechnic University, Guayaquil, Ecuador

**Keywords:** Algorithm animation, Augmented reality, Emotions, Dijkstra's algorithm

## Abstract

Algorithm animations are a resource that assists in learning algorithms by visually displaying the behavior of an algorithm at a higher level of abstraction than source code. On the other hand, augmented reality is a technology that allows extending visible reality in a mobile device, which can result in greater emotional well-being for the student. However, it is not clear how to integrate algorithm animations with augmented reality. The article makes two contributions to this concern. On the one hand, we describe an architecture that allows generating interactive algorithm animations, integrating them appropriately in the context of immersive augmented reality. This way the user can watch the source code of the algorithm, augmented with textual explanations, visualizations and animations of its behavior. We illustrate the use of the architecture by instantiating it to the well-known Dijkstra’s algorithm, resulting in an augmented reality tool that generates text, 2D and 3D visualizations. On the other hand, the influence of the tool on the user’s emotions has been studied by conducting an experience with face-to-face and online students. The results show that, with the joint use of augmented reality and visualizations, the students: experienced significantly more positive than negative emotions, experienced more agitation and stimulation than inactivity or calm, enjoyed as much as they expected, and their feeling of boredom decreased during the experience. However, students felt anxiety from the beginning and it increased with the use of augmented reality. The study also found that the face-to-face or online learning model influences emotions and learning outcomes with augmented reality.

## Introduction

Solving optimization problems is a complex task. These problems seek to maximize benefits or minimize costs, i.e., they do not only seek to find a valid solution to the problem but to find a best solution. Optimization is a multidisciplinary topic that can be analyzed from different approaches such as mathematics, operations research, algorithm theory, etc. From the algorithmic approach, there are several solving strategies that assist in designing algorithms to solve this type of problems. These strategies are so relevant that they are a part of the knowledge core in the education of future computer scientists. The curricular guides defined by ACM and IEEE regarding Computer Science [79] establish the study of algorithms as part of its “body of knowledge” and identify it as fundamental knowledge in computer science. Forecasts for the new syllabuses suggest that it will continue being core content in the coming decades [10] (treating this document with caution as it is published as a draft and it is not the final version yet).

Understanding and applying algorithmic strategies is not easy, as previous research shows. Some difficulties in learning algorithms are shared with learning programming, since both subjects go together when someone learns to program. However, algorithm learning has specific difficulties in topics as heterogeneous as complexity analysis [24], computational complexity [28], correctness [48] or sorting algorithms [80]. As a consequence, we find students’ misconceptions on optimization algorithms [84] or, more specifically, on dynamic programming [16, 21] or branch-and-bound [84] techniques.

Unfortunately, there are no unequivocal recommendations on the materials to be used in the study of the algorithms. Traditionally, students have worked with texts, previously in physical format [14, 77] and lately in electronic format [58]. These texts usually state the optimization problem to solve and describe some algorithm that solves the problem. The static presentation of algorithms does not help in understanding their dynamic behavior and therefore their logic. Probably the best known technology to address this problem is the dynamic visualization of algorithms, i.e., algorithm animation [73]. This visualization technique allows watching the dynamic behavior of algorithms, making their abstract behavior visible and therefore concrete to the student. However, this technology requires a change in the learning environment, where the book disappears as a natural, major learning resource and the virtual environment of the computer appears. This shift from the physical object to the virtual object can affect students’ motivation since manipulating objects has a direct effect on the student’s satisfaction and learning outcomes [50].

Augmented reality is another technology that can potentially alleviate the drawbacks of the switch from the physical world of the book to the virtual world of the computer. The term “augmented reality” encompasses various technologies that enrich the physical environment with digital information, coexisting information from both worlds [5]. There are several ways to integrate the physical and virtual realities [36]. On learning algorithms, visualizations and animations of their behavior are probably the most suitable type of digital information.

In principle, the joint use of augmented reality and visualizations for learning algorithms may have positive effects on students’ motivation and emotions. Thus, some studies on visualization systems have detected improvements in students’ motivation [86] and emotions [51]. Likewise, the use of augmented reality can also increase the motivation, commitment and satisfaction of students with learning activities [38]. Characteristics of augmented reality such as sensory immersion, the sensation of being present in the scene that unfolds and the absorption capacity experienced by the user, significantly influence satisfaction, knowledge and understanding, and therefore has positive learning effects [50].

However, augmented reality can also affect negatively the emotional state of students. Users of the game Pokemon Go were frustrated when some features of their smartphones, such as speed, were reduced by the augmented reality resources used by the game consuming too much battery [60]. Other problems that may appear are related to the user experience. For example, Ibáñez et al. [38] reported that some users experienced usability problems when trying to get the system to recognize the markers, as well as problems when simultaneously manipulating real objects and the mobile device. Other experiences indicate that too much information on the device screen can be negatively perceived by the user [22] and there may be loss of concentration when the user has to read extended information on the screen [38]. These limitations suggest the need to deepen our understanding of the effect of augmented reality on the emotions experienced by the user [18].

The goal of our research is to investigate the joint use of visualizations and augmented reality to learn algorithms, both from the point of view of its technical feasibility and its emotional and learning effect on students. For the study, we have selected a task for the algorithmic strategy known as the greedy technique [14, 43, 70], more specifically a task aimed at understanding Dijkstra’s algorithm.

The article has two main contributions. First, an augmented reality architecture has been designed to generate 3D visualizations and animations of algorithms combining them with 2D representations and textual explanations. Using this architecture, the DARA app (Dijkstra’s Augmented Reality Animation) was developed. Second, an evaluation of the variation of the emotions experienced by the students was conducted. As a result, evidence was found of the influence of the combined use of augmented reality and algorithm animations on the emotional state of the student, identifying both positive and negative effects.

The structure of the articles is as follows. The article begins with a review of the different topics addressed by the article. Section 3 shows the DARA tool and its underlying architecture, designed to support both augmented reality and algorithm visualizations. Section 4 describes the evaluation conducted with students to assess the tool impact. In Sections 5 and 6, the results obtained are presented and discussed, respectively. The article ends with the major conclusions of the research.

## Background

This section presents a review of emotions in education and the impact of augmented reality and visualizations on them. The issues discussed have not always been studied for algorithm learning, so we sometimes present some studies oriented to learning programming, given their affinity.

### Motivation and emotions

Emotions are present in many aspects of our lives and they are a fundamental factor in education [26, 64, 69]. Although it is difficult to define precisely “emotion”, it can be understood as a complex set of interactions with subjective and objective factors mediated by a hormonal/neural system [44].

Emotions are a complex construct, but they can be roughly classified into positive and negative emotions, as in the PANAS questionnaire (Positive And Negative Affect Schedule [82]). Positive emotions are related to enthusiasm, activity, and alertness, whereas negative emotions are linked to anger, contempt, disgust, guilt, fear, and nervousness.

Some authors have addressed the identification of basic emotions, but there is no agreement on them [13]. Some authors propose a small set of basic emotions (between five and eight), with a certain consensus on a minimum of five: fear, anger, sadness, joy and disgust. Other authors prefer to deal with two or three dimensions, in which the intensity of the emotion is expressed. There is consensus on the two dimensions of arousal (from calm to excited) and valence (from attraction to aversion), although two dimensions are insufficient to distinguish some emotions from each other.

More specifically, emotions have been classified in the educational context using the AEQ (Academic Emotions Questionnaire) self-report instrument taxonomy [61, 65]. It is based on Pekrun’s model, which classifies emotions into three dimensions [66]: *object* focus (related to the success and result of activities), *valence* (pleasant or unpleasant), and *activation* (agitation or excitement). Let’s look at the latter two dimensions, as they elicit greater acceptance.

According to the dimension of activation that the subject can experience, emotions can be classified into activation emotions (emotions that produce a high degree of agitation, such as fear, anxiety, anger, etc.) or deactivation emotions (those that produce low agitation, such as depression, calm, boredom, etc.). Likewise, in the dimension of valence, emotions in the academic context can be classified as positive (pleasant sensation) or negative (unpleasant or uncomfortable sensation).

Together, the emotions measured by AEQ are classified as follows [66]:

- Positive emotions with activation: enjoyment, hope and pride.
- Negative emotions with activation: anger, anxiety and shame.
- Negative emotions with deactivation: despair and boredom.

The role of emotions in learning has not been sufficiently studied so far. However, it has received increasing empirical and theoretical attention in recent years, suggesting that emotion plays both a positive and a negative role in the learning process [39, 54, 68]. Thus, when the student manages emotions effectively, he/she can reduce learning time and consequently improve his/her performance [3].

Students who have positive emotional reactions to learning exhibit enhanced abilities to achieve successful outcomes, to develop higher problem-solving skills and are more engaged with the learning experience [65]. Furthermore, positive emotions promote the construction of knowledge in the learner and favor the development of their problem-solving capacity [81]. Park et al. [62] conducted a study on multimedia content and found that students who showed a positive emotional state before starting the learning task obtained better results in the assessments of understanding, transfer and application of knowledge. Therefore, ‘a goal of teaching [should be] to enhance the students’ pleasant achievement outcomes’ [27].

On the other hand, there is a debate about the effect of negative emotions on learning [26], with some authors arguing that negative emotions are a negative factor to avoid, and others arguing that negative emotions can increase student’s motivation. Negative emotions could promote greater attention in the learning environment, generate greater cognitive activity and processing of information in greater detail, generating better learning outcomes [47]. However, negative emotions are generally held to be detrimental to the pursuit of achievement goals, investment of effort, cognitive processes (such as attention and memory), motivation, self-regulation and self-efficacy.

We do not know of works on emotions in algorithm learning, except for one study [85], which assesses the effect of using an interactive experimentation tool on students’ emotions. It was found that students in the face-to-face group experienced more intense emotions than those in the online group. Likewise, all the students decreased their negative emotions, but the students in the face-to-face group also significantly increased their positive emotions.

We find a higher number of related works regarding programming education and students’ emotions [4, 45, 53]. Students’ emotional reactions are often related to the frustration of dealing with the difficulties that are faced to solve programming problems. For example, Bosch et al. [8] found that the emotional states of novice learners of the Python programming language varied as a function of the student’s behavior in the different phases of program construction (design phase, coding, debugging, etc.). The different emotional states experienced during their learning process influenced the results. The relation between students’ positive and negative experiences and their subjective self-efficacy assessment based on interviews has also been studied [45]. Although positive and negative experiences usually occurred with their respective positive or negative self-efficacy assessments, it was found that some students who had a negative programming experience could maintain a positive self-efficacy judgement, while students who had a positive programming experience maintained a negative self-efficacy assessment.

Emotions can have a strong impact on students’ performance, as they can directly cause to fail an exam and even to drop out a course [40]. Some experiences suggest that emotions together with the student’s perception of their ability may have an impact on learning outcomes in introductory programming courses [53]. Zhu et al. [87] developed a C programming online course using the Moodle platform and found a higher correlation between better scores and students with a positive emotional state during the course than for students who experienced negative emotions.

### Programming learning with augmented reality

Education is a prominent application area of ​​augmented reality [1, 6]. Augmented reality has been used educationally in numerous domains, from surgery to engineering. It is less frequent to find it in the teaching of computer science, perhaps because its object of study is digital, thus it makes less sense augmenting the reality with digital information. However, there are interesting and imaginative applications for learning to code.

Some augmented reality applications designed to learn programming use a block-based visual approach. Glenn et al. [30] provides an interaction similar to the visual language Scratch, where users develop programs by drag-and-dropping blocks in a scenario. Their system uses a plug-and-play electronic board and allows interaction with real objects in the story by combining a smartphone to display virtual characters, which carry out actions programmed by the student through blocks. A similar approach is used in robots with AR Bot [59]. Fragments of already built programs are provided and visualized when the user focuses with his/her mobile device on playing cards or physical letters with drawings of robots. Thus, he/she is able to edit and complete those programs and watch virtually the robot behavior in real time. Other applications propose accomplishing simple tasks of finding paths in labyrinths [17].

Other augmented reality tools exploit collaborative learning and gamification for programming [32]. ARQuest [29] provides a physical game board with markers where the main character moves around the board to overcome certain challenges, whose movements must be programmed collaboratively to reach the goal, allowing students to learn the basic concept of sequencing. Other works have focused on the development of serious games, intended to assist in understanding code flow control structures [28] or the logics of event programming [46]. In these collaborative environments, students highly value working as a team, by sharing devices, tasks and knowledge [12, 41].

An important advantage of using augmented reality is the motivation it exerts on the student, generating a high level of satisfaction and enjoyment [30, 56, 72]. Although positive results are not always obtained [17], the characteristics and the ability of augmented reality to visualize information activate the level of attention of the student [46], motivating him/her in the learning task and generating a feeling of interest and participation [12, 25, 29, 30]. This motivation may be greater thanks to the use of gamification resources, such as badges or achievements [28]. In some experiences, students have even continued using the educational resource after completing the educational task [78].

In summary, there is evidence of the influence of augmented reality on motivation for learning programming concepts. Given the relationship between motivation and emotions [23], improvements in motivation lead to the hypothesis that it can also influence positively emotions experienced during learning. However, as far as the authors know, there are no studies on the influence of augmented reality on the emotional state of the student on accomplishing algorithm tasks.

### Learning algorithms with visualizations

Algorithm animation is an area of ​​active research since the 1980s. The student is presented with visualizations of the status of the algorithm execution, which is updated as the execution steps forward (or backwards) for certain input data. Visualizations usually have a higher level of abstraction than the source code, omitting details which are irrelevant to understand the logic of the algorithm. This basic scheme admits different variants from an educational point of view [57]. For instance, the learner may have more or less control over the direction and granularity of the animations or may enter his/her own input data.

There are studies that confirm that the visualization of algorithms improves learning effectiveness under certain conditions, being the educational use of animations more important than their content [37]. Therefore, the student must have an active role, having being proposed a taxonomy of levels of engagement [57]. Very few studies have found negative effects on learning from using visualizations. Crescenzi et al. [15] found that the use of algorithm visualizations requires the student to attend to the execution of the algorithm while not paying attention to the theoretical foundations.

Some studies have addressed questions other than learning effectiveness. Ebel and Ben-Ari [19] studied the effect of visualizations on students’ attention, detecting that their unruly behaviors disappeared. Velázquez et al. [86] analyzed the effect of using the SRec visualization system instead of a conventional IDE for an activity to eliminate recursion, identifying statistically significant higher levels of two components of motivation (intrinsic and extrinsic motivation via identified regulation) in the students who used visualization.

Some studies have measured the variation in student’s emotions, obtaining diverse results. While Haaranen et al. [33] designed educational materials inspired by emotional designs without finding significant improvements in learning outcomes, Lacave et al. [51] detected a decrease in negative emotions in students who used visualizations.

## The DARA tool

DARA has been designed to facilitate learning Dijkstra’s algorithm. The application captures the specific Java source code of the book [70] through the smartphone camera and incorporates text explanations and visualizations of its behavior. In addition, it allows tracing the behavior of the code.

Dijkstra’s algorithm is one of the best-known algorithms of the greedy technique. This algorithmic strategy solves a problem in stages, so that at each stage a candidate is chosen and incorporated into the solution, without being able to reverse the choice subsequently. Explanations of the strategy are often supported with a very high-level scheme, where the elements are represented by auxiliary functions (see Fig. 2 in Section 3.1) [9]. Indeed, at each stage a candidate is selected (*selection function*) and it is decided whether it is feasible to introduce it into the solution (*feasible function*). The algorithm ends when it finds the solution *(solution function*) or no candidates remain.

Although this general, greedy scheme is simple, its application to concrete problems is not evident [83]. In some cases, all the functions that appear may not be necessary or they may even be merged. For instance, Dijkstra’s algorithm selects at each stage a candidate node that minimizes the partial paths from the source node and updates the paths from this candidate node to the remaining candidates, doing this until all node candidates are achieved. In this case, the information on candidates is not known in advance, therefore the feasible function is merged with the selection function, and a solution function is not necessary.

This section describes the main functional aspects of the tool, its design and interaction with the user, as well as its architecture.

### Interacting with DARA

The use of the tool begins by displaying the problem statement and an explanation of the greedy scheme. Subsequently, the interaction proceeds in two phases.

**Fig. 1:**
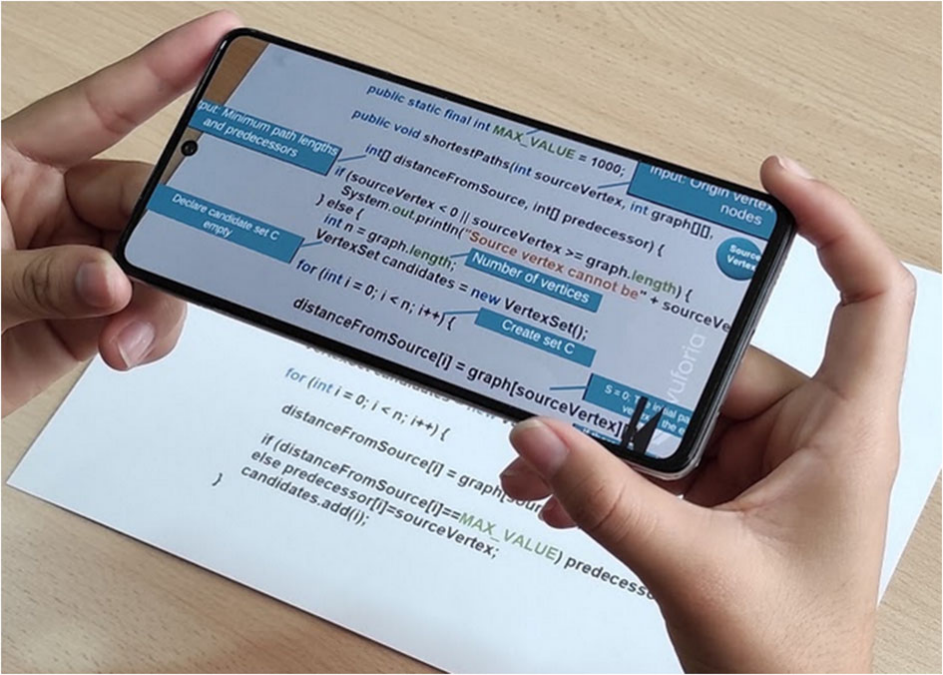
Source code extension with textual information

In a first phase, the smartphone camera is activated and allows focusing Dijkstra’s algorithm source code in Java printed on the book or other physical or digital medium. In order to be concrete, we used the source code of Dijkstra’s algorithm, as included in Sahni’s textbook [70]. Depending on the code fragment focused at each moment, two possible visualizations can be activated automatically. The first visualization are several textual explanations that the tool inserts in the display of the source code captured by the camera (see Fig. 1). These explanations help the user in interpreting and identifying which pieces of source code perform certain functions of the greedy schema.

**Fig. 2:**
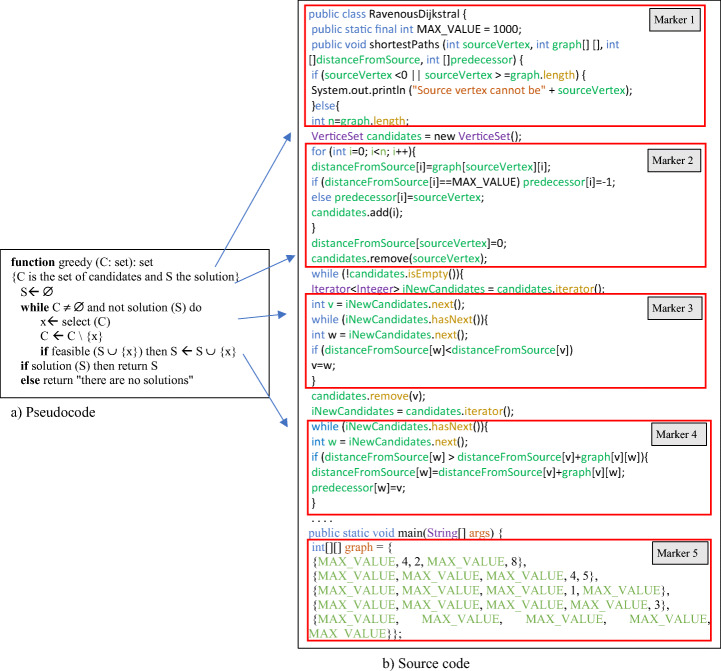
Definition of the markers and relation of the scheme with the algorithm

The objective of this part of the application is to facilitate the understanding of the correspondence between the elements of the greedy scheme (Fig. 2) and those of the source code of Dijkstra’s algorithm (Fig. 2). To support this first phase of DARA use, five augmented reality markers have been defined, as shown in Fig. 2 (markers 1–5). The markers are specific for Dijkstra’s algorithm, therefore creating new markers is required to apply DARA to other algorithms.

**Fig. 3:**
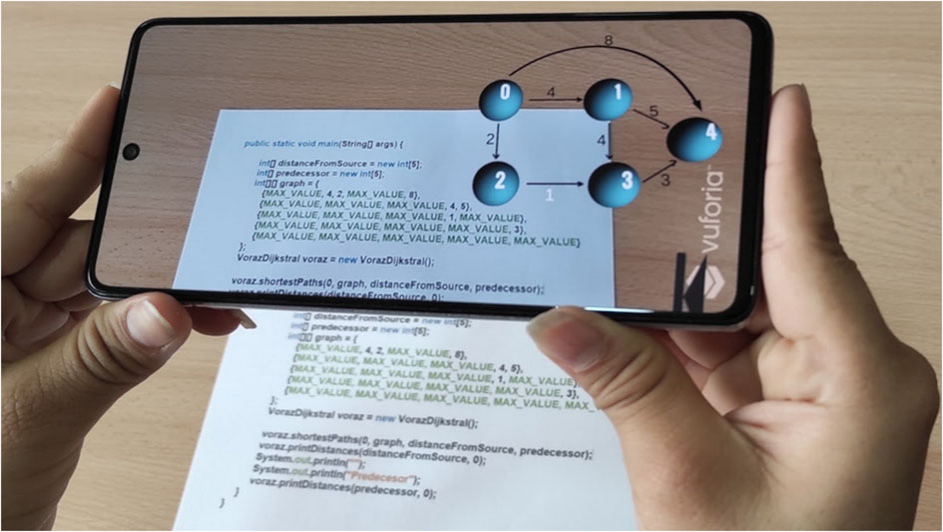
Extending the declaration of the graph with a 3D visualization

The user will move the smartphone camera through the source code reading the augmented text shown by the tool. If the snippet of code captured by the camera is the declaration of an adjacency matrix, the 3D visualization of the corresponding graph is generated (Fig. 3).

**Fig. 4:**
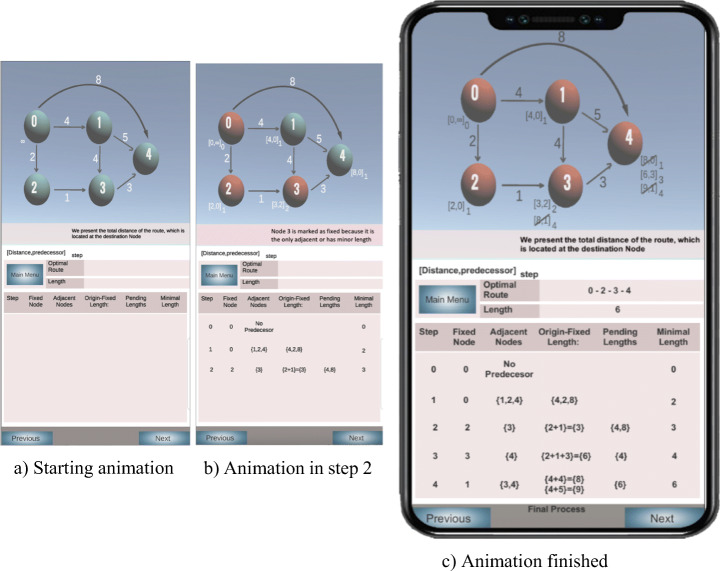
Viewing a 2D animation in several moments

**Fig. 5:**
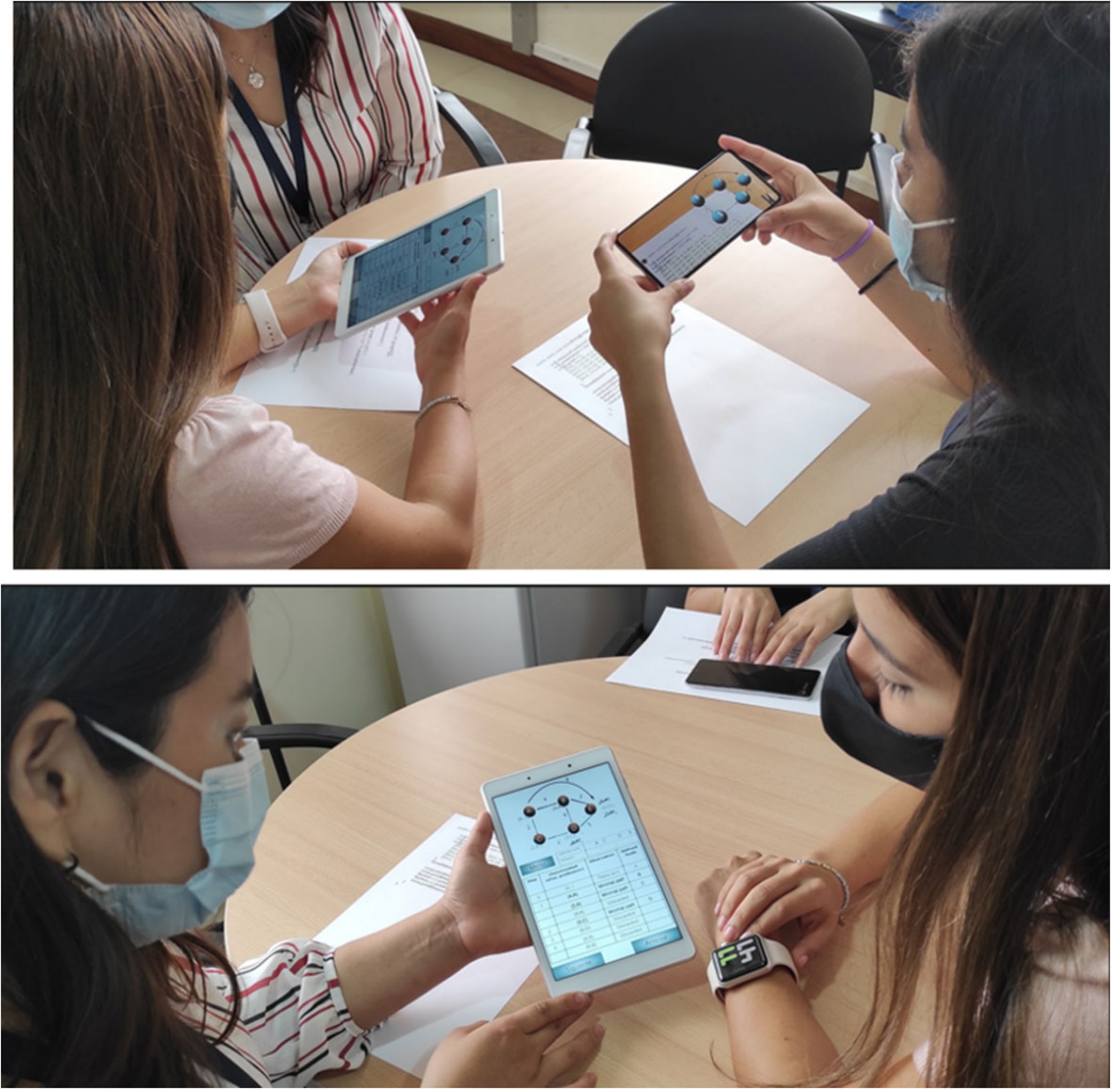
Students using DARA

In a second phase, Dijkstra’s algorithm is applied to a constant, built-in data set. The tool generates a trace of the algorithm and presents a 2D visualization of the previously captured graph, complemented with a table containing the main variables of the algorithm. This screen (Figs. 4 and 5) presents an animation of the algorithm, where the user can trace the algorithm forward or backward at his/her own pace (using the “Previous” and “Next” buttons shown at the bottom of Fig. 4) to better understand the algorithm. This interaction with the animation corresponds to a “controlled viewing” engagement level [55, 76].

The display consists of two parts. The top part shows the graph, with additional information encoded into nodes color and edge labels. Node color allows differentiating the nodes that have been or have not been selected by Dijkstra’s algorithm (in red and green, respectively). Each node also is attributed with one or several labels which encode the minimum distance from the source node, the antecedent node in such a minimum-length path, and the step of the algorithm when such antecedent was found. Note in the figure that all node labels are preserved in order to better understand the building process of minimal-length paths. In this case, we follow the recommendation of incorporating the history of the algorithm into its animation [31].

The lower part of the display shows the evolution of the algorithm in a table with of the most relevant variables used by Dijkstra’s algorithm. The leftmost column contains the number of each step, allowing the user to relate table and node labels information, i.e., the visualization supports the “multiple views” feature [31], working the first column as a coordination mechanism between the graphical and the tabular views.

### System architecture

**Fig. 6:**
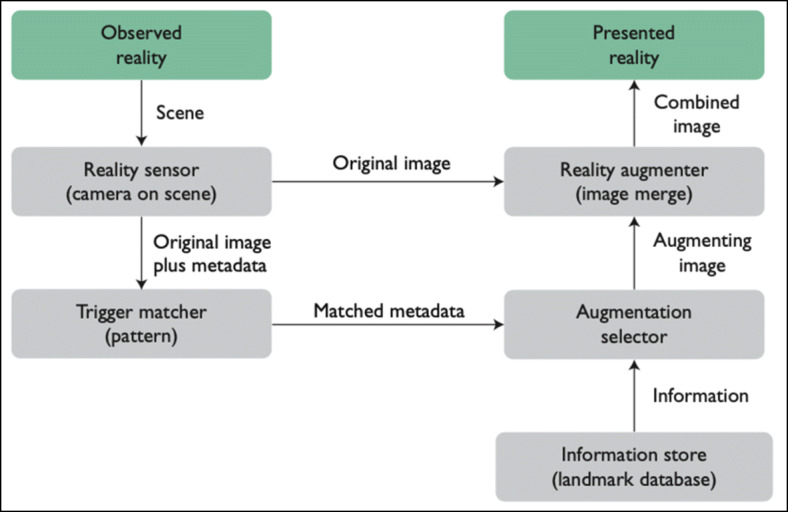
Conceptual architecture of augmented reality proposed by Singh and Singh [75]

The architecture of the system is based on conceptual architecture of augmented reality applications proposed by Singh and Singh [75] (Fig. 6). A reality sensor (camera) observes the reality and passes the image obtained with metadata. Then, the Augmented selector integrates information from landmark database and Reality augmenter combines that information with the original image and renders them for the user. The architecture proposed in the article extends the conceptual model of Singh and Singh integrating a generator of animations and graphic components.

**Fig. 7:**
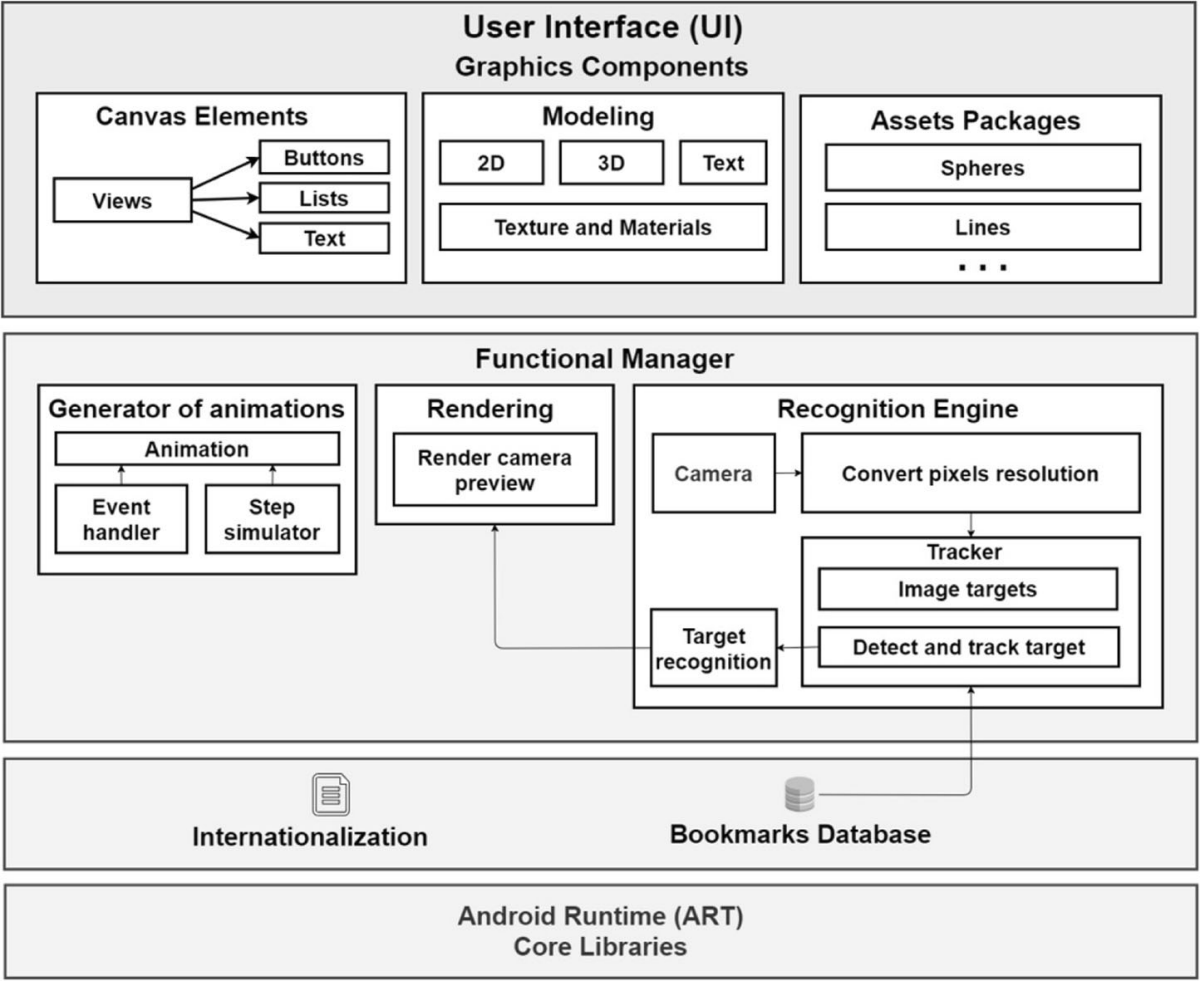
General architecture of the system

The architecture of the system consists of 4 layers (see Fig. 7), which are explained top-down. The first layer is the user interface (UI), which is responsible for displaying the graphic components: different Android views (buttons, text boxes, lists, etc.), the models that are shown by capturing the AR markers (2D, 3D and text visualizations), each with their material and texture properties, and the assets that complement these models, which depend on the type of animation.

The second layer is the Functional Manager, which contains three modules. The module Generator of Animations simulates the algorithm and listens to interaction events aimed at animating the algorithm (advance, pause, go back, etc.), and makes invocations to the layer of Graphic Components, which generates the corresponding visualizations of the algorithm. The Recognition Engine module, developed with Vuforia^1^ technology, captures the markers and decides at all times which model should be displayed. This process is carried out by activating the camera, in such a way that each frame captured and properly converted is transferred to the Tracker, which is in charge of identifying which marker has been recognized by consulting the Bookmarks Database and determining the corresponding target. Finally, the Rendering module adjusts the camera preview, being ready to add the augmented reality objects.

The third layer includes the bookmarks database and the internationalization file that adapts to the language configured on the user’s mobile device. Finally, the fourth layer is made up of the virtual machine (ART) and the Android base libraries, which guarantee the functionality of the platform.

This architecture allows constructing new AR applications to support other algorithms by adapting three parts. Firstly, a simulation of the new algorithm must be implemented within the module Generator of Animations, making its corresponding invocations to the level Graphic Components to generate the visualizations. Secondly, new assets packages must be integrated into the Graphics Components level. Finally, new markers must be included in the Bookmarks Database. For instance, if a new app was to support the knapsack problem [9, 14], the following elements must be developed: a simulator of the greedy algorithm, assets to display rectangles (representing objects and the knapsack), and new markers.

**Fig. 8:**
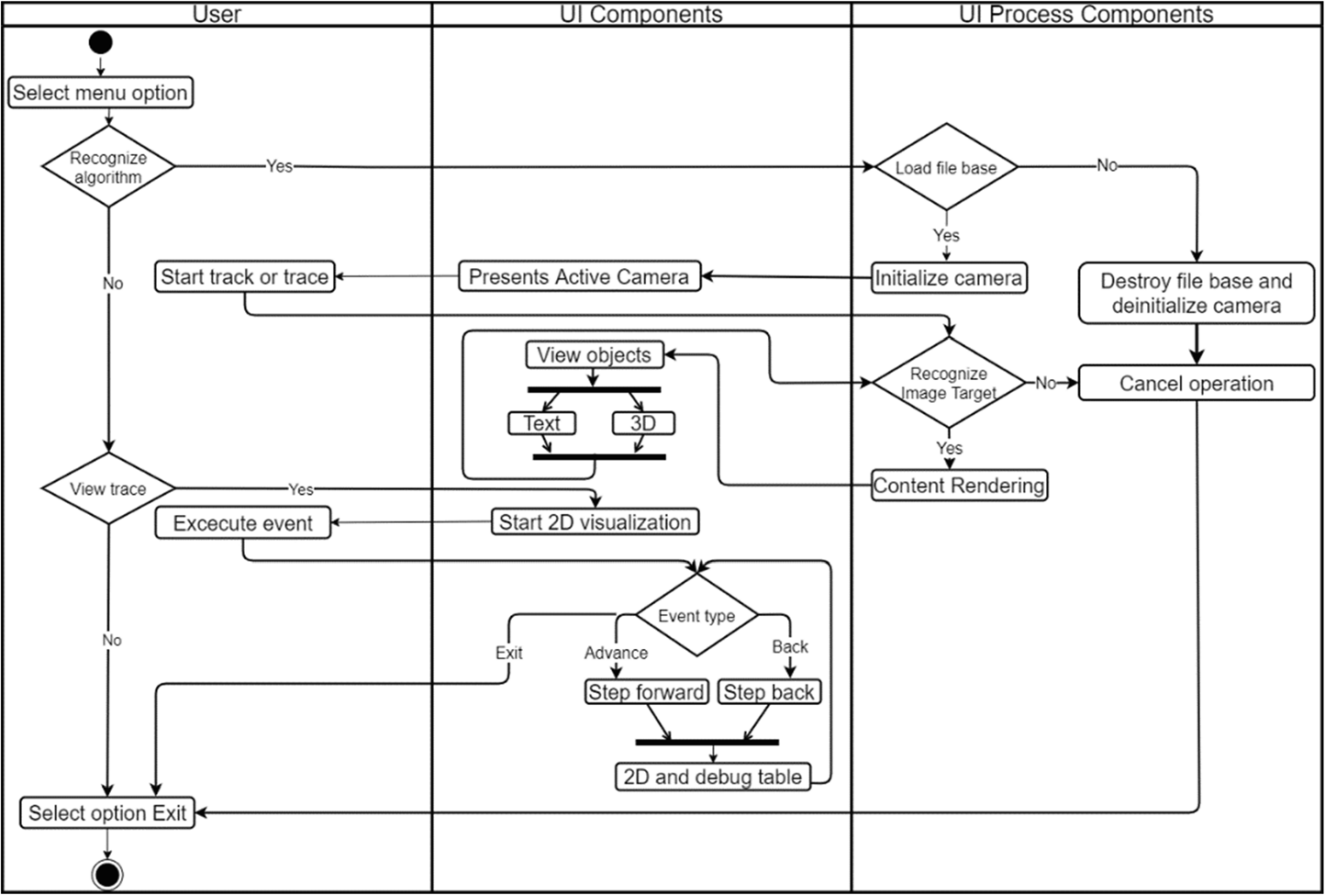
Diagram of activities in user interaction

This architecture supports the use of augmented reality and user interaction. The interaction is triggered from options on a menu provided by the user interface (UI). Figure 8 shows an activity diagram of the two most important interactions where augmented reality and algorithm visualizations are exploited: capturing the source code that has to be expanded with augmented reality and the execution and trace of that code. The activity diagram is divided into three columns (Fig. 8): events that the user performs, the UI components that are generated from those events, and the processing that is triggered accordingly. If the user chooses the option to recognize source code (“Recognize algorithm” in the diagram), the mark base is loaded and the user’s camera is initialized to search for markers, generating virtual objects such as text or 3D, according to the recognized marker. This action is being performed while marks are being recognized. If the user chooses the option to trace the execution of the algorithm (“View trace” in the diagram), the 2D animation will be generated and will be updated according to the user’s event control (forward or backward).

## Evaluation

This section presents an evaluation of the impact of the DARA tool on students’ emotions.

### Hypothesis

An initial objective of the evaluation was to determine the emotions that students experience with the use of the augmented reality tool DARA while they learn Dijkstra’s algorithm. As we have shown in the Section 2, a relevant concern nowadays also is the influence of the specific teaching model used (either online or face-to-face), especially after the COVID-19 pandemic. Therefore, two hypotheses emerge:

- *H1: The positive emotions experienced by the user while using augmented reality to understand Dijkstra’s algorithm are significantly higher than the negative emotions.*
- *H2: The online teaching model with augmented reality significantly improves the emotions and learning outcomes of the Dijkstra’s algorithm compared to the face-to-face teaching model.*

Taking into account the taxonomy of emotions of the model of Pekrun et al. [65], hypothesis H1 can be divided into two sub-hypotheses:

- *H1.1: The positive emotions experienced by the user while using augmented reality to understand Dijkstra’s algorithm are significantly higher than the negative activation emotions.*
- *H1.2: The positive emotions experienced by the user while using augmented reality to understand Dijkstra’s algorithm are significantly higher than the negative deactivation emotions.*

Likewise, hypothesis H2 can be divided into two statements to facilitate its validation:

- *H2.1: The online teaching model with augmented reality significantly improves the learning outcomes in Dijkstra’s algorithm compared to the face-to-face teaching model.*
- *H2.2: The online teaching model with augmented reality significantly improves emotions during the learning of Dijkstra’s algorithm compared to the face-to-face teaching model.*

### Participants and task description

Students from the Salesian Polytechnic University of Ecuador participated during the years 2019 and 2020 in the evaluation. They enrolled in the course Data Structures, of the second year of Computer Engineering. In the course, students learn algorithmic problem solving strategies, as well as their design and implementation in Java.

There were 53 participants with ages varying between 18 and 25, most of them being 19 years old. Students belonged to two different class groups, a face-to-face group made up of 18 students and an online group made up of 35. The students participated voluntarily and had no contact or prior knowledge of greedy algorithms, although they knew the Java language.

The task that the participants had to perform was to understand the Java code of Dijkstra’s algorithm by reading it on printed sheets and by using DARA.

### Process

**Fig. 9:**
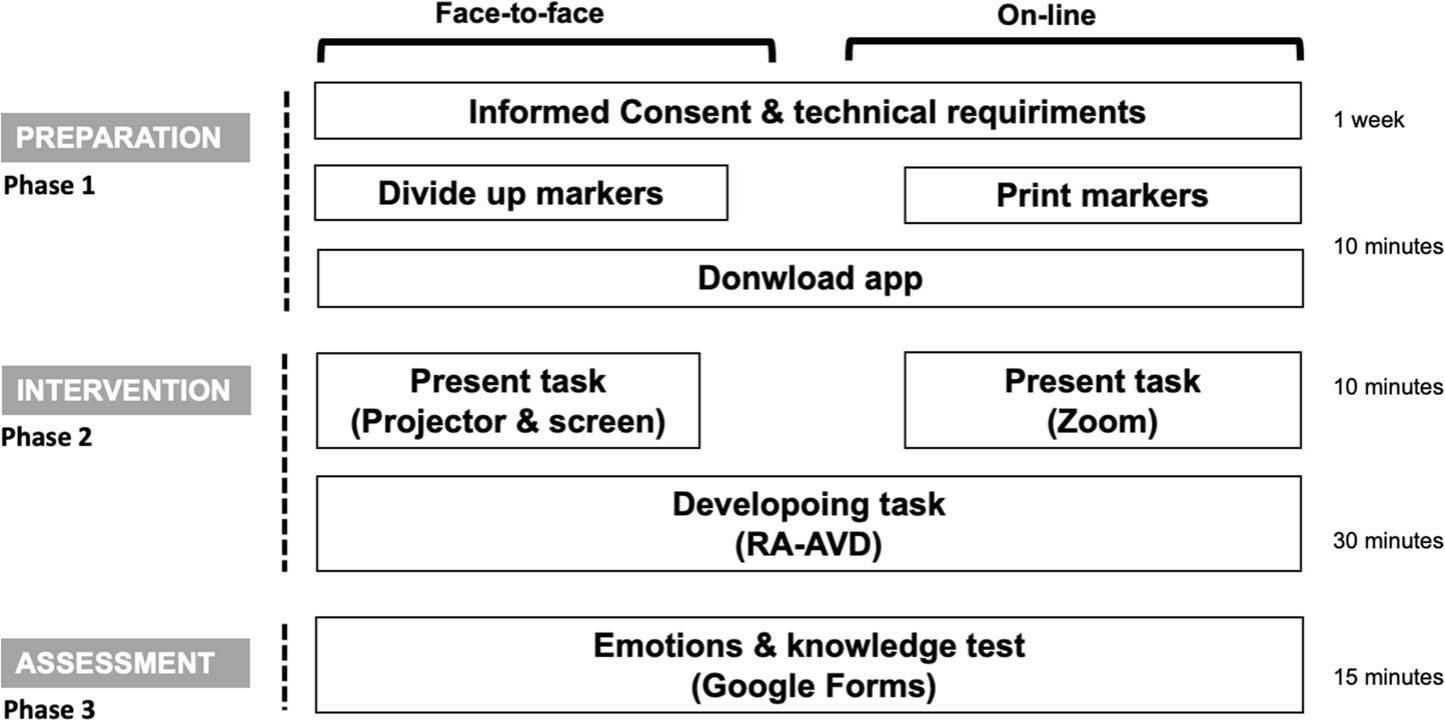
Experience planning

The evaluation varied slightly in the two groups of participants: a face-to-face group, participating in a computer laboratory, and an on-line group, participating remotely from their homes, using Zoom as a tool for video conferencing. The evaluation was conducted in three phases (see Fig. 9):

- Phase 1, preparation. The necessary material was prepared and everything was arranged to be able to begin the intervention. In the first place, the participants were sent a communication through the AVAC learning platform where they were asked for their informed consent to participate and were informed of the activity and the requirements of the mobile devices they would need: Android version 6.0 or higher with rear camera. The participants used their own smartphones. In addition, bookmarks were either provided to the members of the face-to-face group or emailed to the members of the online group, so that they were able to print them. These markers contained the Java source code for Dijkstra’s algorithm (Fig. 2 in Section 3.1).
  The following week the work session was held. The markers were distributed to the face-to-face participants and the online participants were asked to print the markers from their homes and have them ready. Later, all of them downloaded and installed the application through the AVAC virtual platform.
- Phase 2, intervention. The use of the tool and the task they had to perform was briefly explained, using either the slide projector in the classroom for the face-to-face group or Zoom for the online group. Later they began carrying out the task. While reading the Java code on paper, the students could use their smartphones to focus parts of the code and receive help from the application on its operation and simulate its execution. The time allocated for this task was 40 min in both groups (face to face and on-line).
- Phase 3, assessment. In this phase, the knowledge and emotions tests were completed, using 15 min.

The whole experience was organized in a single session and the whole intervention took about 70 min.

### Instruments and variables

The following variables were defined to measure students’ emotions and the level of knowledge they acquired after the experience. As mentioned in the introduction, the authors decided to use the AEQ scale (Achievement Emotions Questionnaire), because the evaluation is carried out in an educational context and AEQ is an instrument validated in an educational context [61]. The AEQ scale measures emotions by offering a series of statements about the participant’s emotional state, which the participant must assess in their degree of agreement by using a Likert scale, from very little (value 1) to extremely (value 5). There are several items per emotion.

The emotional state of the participants with AEQ was measured at three different times:

- Before starting to do the task: the student assesses how he feels just before starting to use the tool.
- During the task: the student assesses how he feels while using the tool.
- After the task: the student rates how he feels after finishing the experience.

Additionally, the effectiveness of the tool for learning was measured. The students filled a knowledge questionnaire on Dijkstra’s algorithm, consisting of 5 multiple-choice questions, where each question scored a maximum value of 2 points. Participants’ opinions about the experience were also collected through with an open question and by observation of students’ reactions during the installation and use of the application.

Consequently, several variables were defined for each emotion, whose names were formed with the name of the emotion followed by the suffix “*_x*” where *x* could be B, D or A, depending on the moment in which the emotion was measured: Before, During or After the experience. Additionally, three variables were defined to calculate the average of positive activation, negative deactivation and negative activation emotions and one more variable to measure the level of knowledge. Table 1 summarizes all the variables measured.

**Table 1:**
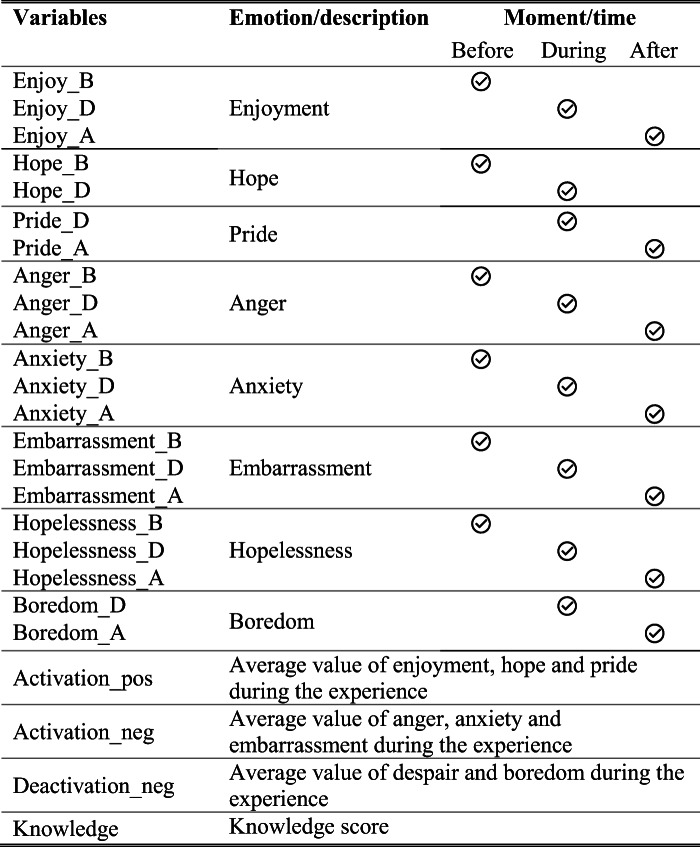
Variables, their definition and collection times

## Results

In a first step, the positive and negative emotions experienced by students from the beginning to the end of the experience were analyzed. Subsequently, an analysis was made that delves into the different emotions and their evolution over time during the development of the experience. Additionally, it was analyzed if there is a difference in emotions and knowledge due to the development format of the experience, either face-to-face or online.

### Study of positive and negative emotions

Table 2 shows the descriptive statistics of the variables involved, where it can be seen that the mean of the Activation_pos variable is greater than the mean of Activation_neg and Deactivation_neg. In order to validate whether this difference is significant, the distribution of these variables was first analyzed, applying the *Komogorov-Smirnov* test (with the *Lilliefors* significance corrections). The variables Activation_neg and Deactivation_neg follow a normal distribution (marked in bold in Table 2) while the variable Activation_pos does not follow a normal distribution. Since there are variables that do not follow a normal distribution and they are all ordinal variables, the *Fridman* non-parametric test for related samples was calculated in order to be able to contrast the equality of means. Table 3 shows the three possible pairs of comparisons, indicating that there is a significant difference, at a 99% confidence level, between the three variables, taking into account the *Bonferroni* correction for multiple comparison.

**Table 2:** Descriptive statistics and validation of the normality distribution of the samples

Variable	N	Mean	Standard Deviation	Variance	Kolmogorov-Smirnov
Activation_pos	53	4.05	0.63	0.398	0.037
Activation_neg	53	2.13	0.17	0.029	**0.200**
Deactivation_neg	53	1.78	0.21	0.042	**0.068**

**Table 3:** Equality test of means

Sample 1 - Sample 2	Statistician	Fridman Test (Sig.*)
Activation_pos-Activation_neg	1,000	0.000
Activation_pos-Deactivation_neg	2,000	0.000
Deactivation_neg-Activation_neg	1,000	0.000

* Adjusted with Bonferroni correction in various tests

### Specific emotions

Each individual emotion was analyzed before, during and after the experience of using the tool. In detail, the variables of the activation positive emotions enjoyment, hope and pride were analyzed, as well as the negative activation emotions of anger, anxiety, and embarrassment, and the negative deactivation emotions of hopelessness and boredom. Each of these emotions was measured at different times through several variables (see Table 1 in Section 4.4). Table 4 shows the descriptive statistics of these variables and the verification of whether they follow a normal distribution (sig.> 0.05), those that do are marked in bold.

**Table 4:** Descriptive statistics of the emotion variables at different times and normality distribution

Variable	N	Mean	Standard Deviation	Variance	Kolmogorov-Smirnov
Enjoy_B	53	4.23	0.97	0.948	0.000
Enjoy_D	53	3.81	0.70	0.496	**0**.200
Enjoy_A	53	3.91	0.86	0.746	0.009
Hope_B	53	4.24	0.70	0.485	0.001
Hope_D	53	4.21	0.73	0.527	0.001
Pride_D	53	4.08	0.71	0.502	**0.076**
Pride_A	53	3.91	0.97	0.943	0.003
Anger_B	53	1.72	0.83	0.694	0.000
Anger_D	53	1.54	0.70	0.487	0.000
Anger_A	53	1.74	1.04	1,083	0.000
Anxiety_B	53	1.83	0.91	0.832	0.000
Anxiety_D	53	2.30	0.80	0.639	**0.200**
Anxiety_A	53	3.28	1.11	1,236	**0.052**
Embarassment_B	53	2.51	1.44	2,062	0.000
Embarassment_D	53	2.25	1.04	1,072	**0.085**
Embarassment_A	53	2.30	1.00	1,010	0.025
Hopelessness_B	53	1.84	1.07	1,142	0.000
Hopelessness_D	53	1.69	0.76	0.584	0.000
Hopelessness_A	53	2.14	0.86	0.732	**0.089**
Boredom_B	53	1.98	0.91	0.836	0.000
Boredom_D	53	1.79	0.71	0.504	0.015

Taking into account the results of *Kolmogorov-Smirnov*, the *t-Student* test was applied to validate the variation of the means in the pairs of variables that followed a normal distribution. Table 5 shows the results of this test where the pairs of variables that are significantly different are marked in bold with a 95% confidence level. You can see that the couple Anger_D-Anger_A is significantly different according to the *t-Student* test, but it ceases to be significant due to the *Bonferroni* correction for multiple comparison (0.046 > 0.05/3).

**Table 5:** Testing variation of the means with *t-Student*

Variables	Statistical	Sig.
Hope_B-Hope_D	0.400	0.691
Boredom_B - Boredom_D	2,175	**0.034**
Anger_B - Anger_D	1,789	0.079
Anger_B - Anger_A	-0.130	0.897
Anger_D - Anger_A	-2,045	0.046

For the rest of the variables that do not follow a normal distribution, the *Friedman* and *Wilcoxon* test was applied for related samples, which rejected the null hypothesis (the means are equal) in the cases of enjoyment, anxiety and hopelessness. Pairwise multiple comparisons were applied to detect which variables are different, taking into account the *Bonferroni* correction for multiple tests. Table 6 shows the results of this comparison where the pairs of variables that are different are marked in bold with a 99% significance level.

**Table 6:** Comparison between pairs using *Friedman* test

Sample 1- Sample 2	Statistical	Sig.*
Enjoy_D-Enjoy_A	-0.340	0.241
Enjoy_D-Enjoy_B	0.792	**0.000**
Enjoy_A-Enjoy_B	0.453	0.059
Anxiety_B-Anxiety_D	-0.623	**0.004**
Anxiety_B-Anxiety_A	-1,500	**0.000**
Anxiety_D-Anxiety_A	-0.877	**0.000**
Hopelessness_D-Hopelessness_B	0.208	0.856
Hopelessness_D-Hopelessness_A	-0.811	**0.000**
Hopelessness_B-Hopelessness_A	-0.604	**0.006**

* Adjusted with Bonferroni correction in various tests

### Classroom and online teaching model

In this section, possible differences in emotions and knowledge between the participants at the end of the experience are analyzed, broken down by their membership in the face-to-face group or the online group. Only the variables that measured emotions and knowledge at the end of the session were analyzed (i.e., variables ending with the suffix “_A” or otherwise “_D”). The *Shapiro-Wilk* normality test was applied, since the samples were less than 50 (18 participants in the face-to-face group and 35 in the online group) and consequently the *Mann-Whitney* and *t-Student* tests were calculated. Table 7 shows the results of this analysis where they are marked in bold in the column “Sig. U of Mann-Whitney/t-Student”. The significant differences found in Activation_neg, Deactivation_neg and Knowledge variables.

**Table 7:** Comparison and contrast of means by groups (FG = Face-to-face Group/OG = Online Group)

Variable	FG’s mean (N = 18)	OG’s mean (N = 35)	Shapiro-Wilk (PG / OG)	Sig. (U Mann-Whitney/t-Student)
Enjoy_A	3.70	4.01	0.545 / 0.001	0.247
Hope_D	3.96	4.33	0.187 / 0.000	0.059
Pride_A	3.67	4.03	0.051 / 0.001	0.383
Anger_A	1.83	1.69	0.000 / 0.000	0.472
Anxiety_A	3.36	3.24	0.396 / 0.062	0.712*
Emabarrassment_A	2.33	2.29	0.059 / 0.033	0.872
Hopelessness_A	2.24	2.09	0.087 / 0.020	0.623
Boredom_D	1.94	1.71	0.051 / 0.002	0.232
Activation_pos	3.82	4.17	0.876 / 0.043	0.091
Activation_neg	2.21	2.09	0.571 / 0.000	**0.000**
Deactivation_neg	2.02	1.66	0.225 / 0.442	0.000*
Knowledge	6.94	5.00	0.068 / 0.004	**0.003**

* t-Student for equality of means at 95% confidence interval

### Correlation between knowledge and emotions

The knowledge acquired by the students at the end of the session was gathered in the Knowledge variable, which had a value of 5.66 (Table 8). The *Kolmogorov-Smirnov* test was applied, confirming that this variable did not follow a normal distribution (*sig.* = 0.000). Consequently, the correlation of the knowledge variable with the emotion variables was calculated using the *Spearman* test. Table 9 shows the correlations found, where three correlations have weak degree (values ​​less than 0.4) and one correlation has medium degree (between 0.4 and 0.6).

**Table 8:** Description of the Knowledge variable and normality test

N	Mean	Standard Deviation	Variance	Kolmogorov-Smirnov
53	5.66	2.78	7,748	0.000

**Table 9:** Knowledge correlation

	Knowledge
Boredom_B	0.387
Boredom_D	0.273
Activation_neg	0.376
Deactivation_neg	0.443

## Discussion

This section discusses the results on emotions obtained both globally and individually throughout the experience. Subsequently, the influence of the teaching model on the emotional state and on the learning results is discussed.

### Influence on positive and negative emotions

The results indicate that the means of positive emotions are higher than the means of activation and deactivation negative emotions, and that this difference is significant. Therefore, we can accept hypotheses *H1.1* and *H1.2*, confirming that the user’s positive emotions while using augmented reality to understand Dijkstra’s algorithm are greater than negatives (both activation and deactivation emotions).

Previous research found similar evidence [18]. Moreover, Khan et al. [42] found that motivation and satisfaction were increased when using augmented reality. Poitras et al. [67] studied the relationship of emotions to user activation when using augmented reality and they found that participants reported higher levels of positive than negative emotions. However, even though they had also used an AEQ-based instrument, these authors were unable to identify the relationship of emotions with activation and deactivation. The study presented here extends this previous research by applying two of the three dimensions of Pekrun et al.’s model of emotions [66], which has facilitated finding evidence that deactivation emotions are significantly lower than activation emotions (both positive and negative). Therefore, we may claim that the participant experiences greater degree of agitation and stimulation during the use of augmented reality than stillness or calm. Although this finding was not established as an initial hypothesis of the present study in this research, its importance must be emphasized since there are no studies to date that have identified this phenomenon for the use of RA in relation to the activation and deactivation stimuli experienced by students. Although the degree of agitation can be due to both positive (e.g., enjoyment) and negative (e.g., anxiety) emotions, it should not be understood as a negative aspect because activation emotions can encourage greater attention in learning tasks and greater cognitive activity, leading to better learning outcomes [47].

### Variations in emotions during the experience

Another important part of the study is the evolution of individual emotions over time, where significant results were found for the emotions of hopelessness, anxiety, enjoyment and boredom. The results indicate that hopelessness and anxiety increased at the end of the task. While hopelessness increased significantly only upon completion, anxiety grew steadily from the beginning of the task to completion. This finding is in contradiction with some previous works. Dirin and Laine [18] carried out an experience where they estimated that 87% of the participants did not consider the experience of using augmented reality unpleasant and only 5% considered it as such. However, Ibáñez et al. [38] found that users experienced anxiety at the end of the RA experience. Our research supports the work of Ibáñez and her colleagues and confirms that anxiety grows throughout the performance of the task with the use of the augmented reality application.

The increase in anxiety may be due to the confluence of two issues. A first issue is interaction with the extended textual information provided by the tool. In the first stage of the task, the student reviewed the source code, thereby interacting with the tool, obtaining textual information that was actually expanded. Textual information can sometimes act as an intrusive component in visual and tactile interaction [38]. In addition, specifically in STEM educational contexts, the student may even evaluate negatively the occurrence of much textual information on the screen [22]. During the experience, some students claimed interaction problems when viewing expanded text since the tool sporadically did not capture the markers in the source code. In fact, some students had to tilt 45 degrees the tables they were working with to make it easier to pick up the markers and display the expanded textual explanations. In summary, the increase in anxiety present from the beginning of the task may be influenced by the high interaction of textual information that the smartphone screen provided to the participant and by interaction difficulties for the Smartphone to detect the expanded information.

A second issue is the complexity of the task. In the second stage of the task, the student must apply the knowledge acquired in the first stage on Dijkstra’s algorithm to a particular problem and try to understand the execution trace that is generated. This increase in the complexity of the task corresponds to a rise from the Understand level of Bloom’s taxonomy to the Analyze level [49], which can generate anxiety. Learning tasks with augmented reality environments that require the application of acquired knowledge can end up generating anxiety in the participants [38]. In summary, the interaction with augmented reality and the complexity of the task may be behind the anxiety experienced by the user.

In relation to the emotion of enjoyment, previous studies have found that the student enjoys learning with the use of augmented reality [52, 63]. However, we have not found clear evidence of this phenomenon: the average enjoyment was initially high (4.23), then significantly lower (3.81) and at the end experienced a rise (3.91). Therefore, the participants expected to enjoy the activity more than they actually enjoyed while they were doing it, although at the end of the experience the feeling rebounded. This discrepancy on the forecast of enjoyment has also been found elsewhere [34], although in a different way. These authors carried out an experiment with two studies using two different augmented reality applications for the same domain, and found that in one study the participants enjoyed more than they anticipated in the pre-test and in the other study they enjoyed slightly less. We consider that the high valuation of the participants in the forecast of what they are going to enjoy may be caused by the high expectations and the novelty of the use of augmented reality. Some users indicated “… it is interesting to learn using an augmented reality application, more than anything because we had never used it” and “we were surprised to learn in this way and more with augmented reality”.

Regarding the boredom experienced by the participants, it should be noted that this decreased significantly when they were doing the task compared to what the participants themselves predicted at the beginning of the task. In addition, boredom was one of the three emotions that they felt with less intensity while carrying out the experience, with a value of 1.79 out of 5. This result is aligned with the works of Dirin and Laine [18], which showed that only 10% of users experienced boredom, and with the investigations of Ibáñez et al. [38], whose authors found that boredom was experienced only in the early stages of the task.

### Implications of the online and face-to-face teaching model

The results indicate that the teaching model used influences both the emotions and the learning outcomes obtained by using of augmented reality. The students were unaware of the greedy strategy as they had not studied it in previous courses of their curriculum. They were also asked, before the intervention, if they knew the greedy strategy, to which they all answered negatively; only two students answered that they had heard of it, but did not know it. As expected, at the end of the experience, there was a learning gain, since the group of students reached an average of 5.66 points out of 10. However, it was found that this learning gain was significantly higher in the face-to-face group (6.94) than in the online group (5.0). Therefore, hypothesis H2.1 is rejected, concluding that it cannot be said that the online model improves learning outcomes compared to the traditional face-to-face model. This learning difference between the two models may be related to the emotional experiences lived.

The analysis of emotions carried out indicates that students in the face-to-face group experienced significantly more negative emotions (both activation and deactivation) than those in the online group. Therefore, hypothesis H2.2 is accepted, confirming that the online teaching model significantly improves emotions compared to the face-to-face teaching model. The authors do not know the reason for this effect since learning task, augmented reality and teachers were the same in both groups. Velázquez and Palacios [85] evaluated the emotions during a session with students broken down into the two conditions, face-to-face and online, finding that the participants in the face-to-face group experienced higher levels of all emotions, positive and negative. Choi et al. [11] state that the physical environment affects the cognitive load and affective state of the student in the context of learning. There are studies that show that specific learning and knowledge assessment environments can generate anxiety in students and affect the ability to perform the task [7]. In our case, the online group obtained significantly lower learning results than the face-to-face group (5.0 vs. 6.94). Therefore, the difference in negative emotions between both groups could be a key factor in explaining the difference in learning outcomes. For instance, the students in the online group, experiencing fewer negative feelings, were able to be more relaxed and make less effort during the task, affecting the learning outcome.

Finally, the study of correlation between the level of knowledge acquired and emotions has not found significant results, finding only weak correlations with negative emotions. These results seem to suggest that the level of knowledge acquired tends to be related to negative emotions. The authors suggest conducting additional studies to deepen into this issue.

### Using algorithm animations with augmented reality

One of the key characteristics of the tool proposed in the article is the ability of the user to interact with algorithm animations. This aspect is unusual at augmented reality platforms for rendering visualizations. Amaguaña et al. [2] propose a platform for military tactical training by detecting flat surfaces with Vuforia. Although it generates animations, these are automatic and do not support interaction. Other architectures can generate simulations, but these are programmed in design time and do not support dynamic interaction during the deployment of augmented reality [74].

Some works have presented augmented reality platforms that support interaction, by either dynamically generating their own visualizations [35] or integrating intermediate resources such as avatars [71]; some platforms even incorporate analytical methods of user interaction to identify behavioral patterns [20]. However, there are no augmented reality platforms that support algorithm visualizations and animations, which have a high degree of complexity in representing the state of the algorithm throughout its execution time. Some augmented reality platforms make it easy to learn programming in introductory courses. In some cases focused on textual programming languages learning [72] or basic data structures learning [56]. But in any case, they do not reach the degree of sophistication necessary to support the learning of optimization algorithms, such as step-by-step interactive control of algorithm or coordination mechanisms between multiple views.

Our architecture is based on Singh and Singh’s work [76], who proposed a conceptual architecture for augmented reality platforms, which includes the combination of metadata of observed reality with stored information to increase the presentation to the user. The architecture of DARA extends this conceptual model by incorporating the animation generation module, which combines the observed reality with stored graphic components to generate animations. Consequently, the present work shows that it is possible to integrate the generation of interactive algorithm animations into augmented reality architectures.

## Conclusion

The article has presented an augmented reality tool with 3D visualizations and animations aimed at understanding Dijkstra’s greedy algorithm. As far as the authors know, this is the first study in which augmented reality is applied to learning optimization algorithms and student’s emotions are analyzed along its use. Typically, the use of augmented reality in computing education is limited to applications with visualizations for learning basic programming concepts, but it does not exploit the use of interactive animations.

The contributions of the article are two-fold. First, a software architecture is provided which combines augmented reality and visualizations, and its instantiation allowed constructing an app aimed at Dijkstra’s algorithm, i.e., the DARA tool. Second, several issues on the impact students’ emotions were measured. Emotions were measured using the validated AEQ scale, developed for educational contexts. The progress over time of students’ emotions while interacting with augmented reality was also studied, as well as whether the face-to-face or online class model influenced emotions and learning outcomes.

The results of the work show that the student experiences significantly more positive than negative emotions during his/her interaction with augmented reality. Throughout the experience, feelings of boredom and enjoyment decreased significantly (although the latter with a final rebound) while anxiety increased throughout the experience. In addition, the results allow claiming that the users experienced significantly more agitation and stimulation than inactivity or calm during the interaction with the tool, thus awakening an effect of activity in the user.

The study has also identified differences between the classroom and online class models. The face-to-face group obtained significantly better academic results than the online group, however, the former experienced more negative emotions than the latter.

Some additional lessons learned during the experience can be pointed out. On the one hand, it is important to have adequate ergonomics to interact with augmented reality. In the face-to-face experience, users observed that they sometimes had problems capturing the augmented reality markers and that by tilting the source code sheet they were better captured, so it was decided to tilt the desks 45º during the session. On the other hand, ambient brightness should not be very high. Light intensity had to be adjusted to avoid reflections on the source code papers and to facilitate the capture of virtual reality markers. In summary, it is advisable to check the classroom conditions, such as furniture and lighting, before the session.

As future work, we consider that analyzing the characteristics of animations and specific features of augmented reality would be very interesting research lines to deepen into our understanding of the joint use of these technologies. Moreover, we want to delve into the effect of negative emotions on academic results. Some studies indicate that negative emotions can improve learning outcomes by increasing cognitive activity and attention to the learning task [47]. We have found certain indications that point to this possible improvement: we found a weak correlation between negative emotions and the knowledge acquired, as well as that students of a group experienced more negative emotions and obtained better results than students of the other group.

## Footnotes

1
https://developer.vuforia.com/.
